# Religious leaders’ perceptions of advance care planning: a secondary analysis of interviews with Buddhist, Christian, Hindu, Islamic, Jewish, Sikh and Bahá’í leaders

**DOI:** 10.1186/s12904-017-0239-3

**Published:** 2017-12-28

**Authors:** Amanda Pereira-Salgado, Patrick Mader, Clare O’Callaghan, Leanne Boyd, Margaret Staples

**Affiliations:** 1 Centre for Nursing Research, Cabrini Institute, 154 Wattletree Road, Malvern, VIC 3144 Australia; 2 Faculty of Medicine, Nursing and Health Sciences, Monash University, Wellington Road, Clayton, VIC 3800 Australia; 3 Palliative and Supportive Care Research Department, 154 Wattletree Road, Malvern, VIC 3144 Australia; 4 Departments of Psychosocial Cancer Care and Medicine, St Vincent’s Hospital, The University of Melbourne, 41 Victoria Parade, Fitzroy, VIC 3065 Australia; 5 Institute for Ethics and Society, The University of Notre Dame Sydney, L1, 104 Broadway, Sydney, NSW 2007 Australia; 6 Australian Catholic University, 115 Victoria Parade, Fitzroy, VIC 3165 Australia; 7 Monash Department of Clinical Epidemiology, Cabrini Institute, 154 Wattletree Road, Malvern, VIC 3144 Australia

**Keywords:** Religion, Spirituality, Faith, End-of-life, Advance care planning, Health professionals, Palliative care, Qualitative, Secondary analysis

## Abstract

**Background:**

International guidance for advance care planning (ACP) supports the integration of spiritual and religious aspects of care within the planning process. Religious leaders’ perspectives could improve how ACP programs respect patients’ faith backgrounds. This study aimed to examine: (i) how religious leaders understand and consider ACP and its implications, including (ii) how religion affects followers’ approaches to end-of-life care and ACP, and (iii) their implications for healthcare.

**Methods:**

Interview transcripts from a primary qualitative study conducted with religious leaders to inform an ACP website, ACP*Talk,* were used as data in this study*.* ACP*Talk* aims to assist health professionals conduct sensitive conversations with people from different religious backgrounds. A qualitative secondary analysis conducted on the interview transcripts focussed on religious leaders’ statements related to this study’s aims. Interview transcripts were thematically analysed using an inductive, comparative, and cyclical procedure informed by grounded theory.

**Results:**

Thirty-five religious leaders (26 male; mean 58.6-years-old), from eight Christian and six non-Christian (Jewish, Buddhist, Islamic, Hindu, Sikh, Bahá’í) backgrounds were included. Three themes emerged which focussed on: religious leaders’ ACP understanding and experiences; explanations for religious followers’ approaches towards end-of-life care; and health professionals’ need to enquire about how religion matters. Most leaders had some understanding of ACP and, once fully comprehended, most held ACP in positive regard. Religious followers’ preferences for end-of-life care reflected family and geographical origins, cultural traditions, personal attitudes, and religiosity and faith interpretations. Implications for healthcare included the importance of avoiding generalisations and openness to individualised and/ or standardised religious expressions of one’s religion.

**Conclusions:**

Knowledge of religious beliefs and values around death and dying could be useful in preparing health professionals for ACP with patients from different religions but equally important is avoidance of assumptions. Community-based initiatives, programs and faith settings are an avenue that could be used to increase awareness of ACP among religious followers’ communities.

## Background

Advance care planning (ACP) promotes consideration of values, beliefs, and health care goals which should be communicated or documented so that an individual’s care wishes are upheld if they become incapable of making informed decisions at end-of-life [1]. International guidance for ACP recognises the importance of integrating spiritual and religious aspects into planning practices [2–4]. Spirituality relates to the ways individuals express meaning and experience their connectedness to others, nature, and that deemed significant or sacred [5]. Spirituality may include religion which denotes beliefs, rituals, and practices related to the sacred, enables closeness with the transcendent, and is practiced communally or privately [6].

Religious beliefs and values can inform future care decisions including whether to make them [7–14], influence how autonomy is valued among different individuals [4], preferences for care [3, 15], and preparedness to deal with the dying process [2]. Quantitative studies examining religion, end-of-life decision-making and ACP found that patients with positive religious coping or greater religious beliefs were more likely to prefer life-extending treatment [9, 10, 16]. Reports on patients’ willingness to engage in ACP are however mixed [17–19].

Strategies promoting ACP in healthcare and community settings are widely evident [2, 4, 20–29]. ACP benefits include improved patient quality of life and satisfaction [30, 31], fewer hospitalisations [31, 32], increased use of hospice and palliative care [31] and improved mood and adjustment in bereaved survivors [33]. There is, however, widespread variation in ACP uptake [34–38]. Worldwide the majority of people affiliated with a religion is expected to grow [39], yet spiritual and religious needs are often inadequately addressed at end-of-life [7].

A recent systematic review reported that physicians inconsistently inquire about patients’ religious and spiritual views and prefer to refer patients to chaplains for spiritual discussions [40]. Difficulties encountered by health professionals in providing spiritual or religious care include inadequate training or education [41–44], limited time [41–44], fear of causing patients discomfort [41, 42] and difficulties in finding appropriate expressions [42]. While health professionals may perceive spiritual support to be the sole domain of religious and faith leaders, health professionals also need to be able to consider patients’ spiritual and religious values in the context of health decision making and care [11, 45, 46].

Religious leaders often provide spiritual support to patients and families at this time [47, 48] through understanding patients’/families’ faith traditions and interpretations about death and dying. Their perspectives on religion’s influence on followers’ approaches towards ACP could illuminate how health professionals may address the “core principle” of supporting spiritual and religious well-being at end-of-life [49] and in relation to ACP.

Qualitative research has focussed on patients’ [50–52], caregivers’ [53], clinicians’ [54, 55], and government officials’ [55] perspectives of ACP. We found only one study of religious leaders’ perspectives of ACP, which revealed that Singaporean Catholic Nuns were familiar with and accepting of ACP [56].

An Australian-based national ACP website, ACP*Talk* [57], was recently developed, intended to support health professionals conducting sensitive ACP conversations with people from selected Australian-based religions (Christian, Hindu, Muslim, Buddhist, Jewish, Sikh, Bahá’í). Website content included information derived through the deductive analysis of interview transcripts collected from religious leaders aligned with the researchers’ predetermined topic areas. The aim of the primary study was to describe: (a) each religion’s doctrine and practices associated with death and dying;(b) their advice on disclosing prognoses; and (c) conducting ACP conversations with the religion’s followers. Within these interviews, religious leaders also outlined broader, unanticipated considerations about ACP related to their faith communities.

Given the paucity of literature regarding religious leaders and ACP, a qualitative, inductive, and exploratory secondary analysis [58, 59] was conducted on the religious leaders’ interview transcripts to address related but different research aims from those which informed the primary, deductive interview analysis and ACP*Talk* website. This secondary analysis aims to examine: (i) how religious leaders understand and consider ACP and its implications, including (ii) how religious leaders consider religion affects followers’ approaches to end-of-life care and ACP; and (iii) their implications for healthcare.

## Methods

### Setting, participants, and procedure of primary study

In the primary study, designed to establish the ACP*Talk* website, religious leaders residing in Australia were invited to participate in semi-structured interviews. Leaders were senior members of a religion with expertise on its teachings on death and dying. Leaders were selected through purposive sampling and snowballing [60], with members of the ACP*Talk* Project Advisory Committee recommending participants.

Following Cabrini Health Human Research Ethics Committee approval (CHREC 02–05–10-15), two trained research assistants, PM and another involved in data collection only (male qualified BHSc; female qualified BHSc/BTh) invited potential participants via telephone or email. The research assistants were trained by the experienced lead clinician researcher APS (female qualified BN, RN, MPH) in obtaining consent, data collection and management procedures. Interested participants were emailed a participant information consent form. Following written consent, face-to-face or telephone interviews were scheduled at mutually convenient times and conducted by research assistants with participants in their home, workplace or in the research organisation’s meeting room (face-to-face only). No relationships with participants were established with any members of the research team prior to the study. Interviews were voice recorded and transcribed verbatim. Repeat interviews were not required and therefore not carried out. Field notes were not made. A demographics questionnaire (age, sex, country of birth and religion), and semi-structured interviews (Table 1) were conducted from November 2015 to February 2016.

**Table 1:** Question interview framework for religious leaders

1. What is your current understanding of advance care planning? *(ACP clarified if needed)*
2. When there is a diagnosis of serious illness, some people prefer to hear about their prognosis and some prefer not to. How appropriate is it in your religion to disclose information about poor prognosis to the ill?
3. When conducting advance care planning conversations with people of your religion, how should health professionals approach the conversation? *(How, when, where, whom, what questions might they ask?)*
4. What particular language or terminology is appropriate or inappropriate?
5. Advance care planning involves decision making around treatment options. For people in your religious group, who should be in involved in decision making? *(family, friends, religious leader)*
6. What festivals or rituals might impact advance care planning conversations (*celebrations, fasting, special times*?)
7. We’d like to hear about the beliefs and rituals around death and dying for people of your religion. What are the particular beliefs? What are the rituals?
8. What other special considerations do health professionals need to be aware of for conducting advance care planning conversations in your religious group? *(age, gender,* etc.*)*
9. Is there anything additional you feel would be important for health professionals to know in advance care planning with people from your religious group?

The primary study aimed to gather an extensive amount of data related to each religion’s doctrine and practices associated with death and dying, advice on disclosing prognoses and conduct of ACP conversations. Resources and timelines were insufficient to enable the extensive data collection needed to potentially saturate findings in relation to all Australian religions. Sufficient information was acquired to inform religious leaders’ views for the purpose of secondary data analysis.

### Secondary data analysis

Interview transcripts from all religious leaders interviewed in the primary study were included in the secondary analysis. Qualitative secondary analysis is a research method whereby existing data sets are used to answer associated but different questions to those examined in an original study [58, 59].

Qualitative data were analysed and reported as per the consolidated criteria for reporting qualitative research (COREQ) guidelines [61], except data analysis was not returned and confirmed by the religious leaders. Arguably, interpretations evolve and cannot be revisited [62]. The analytic approach was a qualitative description design with grounded theory features [63, 64]. Transcripts were thematically analysed using an inductive, comparative, and cyclical procedure. Data segments were coded with researcher created labels, which were grouped into researcher created categories, which were in turn grouped into researcher created themes. CO, an experienced qualitative researcher (female with qualifications BMus, BSW, MMus, PhD), conducted the initial analysis. APS and PM provided qualitative inter-rater reliability [65]. APS and PM read the interviews and CO’s analysis, and then discussed the findings with CO. Findings were adjusted until all reached agreement on their final representation. Qualitative data management software was used [66].

## Results

Thirty-five religious leaders, representative of Buddhist (*n* = 4), Christian (*n* = 16), Hindu (n = 4), Islamic (n = 4), Jewish (*n* = 5), Sikh (n = 1) and Bahá’í (n = 1) faiths were included with a participation rate of 54.68%. Reasons for refusal were: not interested (*n* = 9), cancelled/did not show up (*n* = 2), too busy (n = 9) or unknown (n = 9). Participants were predominantly male (*n* = 26), mean age 58.6 years old (standard deviation [SD] 11.6) with nearly half born overseas (n = 16, 45.7%). Mean interview duration was 33.09 (SD 10.75) minutes.

Three themes and nine category findings emerged. An example of data analysis is illustrated in Table 2. In the following clarification of the themes, participants are described by religious affiliation and participant identification number.

**Table 2:** Four categories, and text and code examples, which informed Theme 2, Religious followers’ diverse approaches towards end-of-life care reflect varied religious and cultural backgrounds, and faith interpretations and attitudes

Text	Codes	Categories
I think the generations are different. Younger people talking about 30 years and younger especially if they’re brought up in Australia have a different approach to handling that situation. The older generation tends to be different. The older generation is a lot more conservative, more faithful I guess. I don’t want to generalise. And more not so proactive, more reactive. Whereas the younger generation I think wants to be a bit more proactive. They want to know the facts and they want to know the truth. Sometimes gender does play a part. Some families are very strong in terms of the gender, the male being the head of the family and the male needs to make decisions. But again, that’s more to do with the older generations. Younger generation are more about equality and being partners in the decision. (Coptic Orthodox 31)	Coptic country of origin differences Generational differences: older male tends to make decisions; elder more conservative & faithful; younger want the truth (Coptic)	2a. Family and geographical origin and backgrounds
Anglican Church is quite strong in parts of Africa and I wouldn’t know how to speak about how Black Africans might or however we approach the culture, but Africans how Africans and different regional groups might want to talk about end of life issues. They would take a cultural position that would have nothing to do with being Anglican or not. There’s quite a lot of Chinese Anglicans and I think the same would be true for them. I think really I can only talk about White Anglo Celtic Anglicans, which are English, Australian, New Zealand, American, probably White South African. Cultural is probably more important than religion in lots of ways. (Anglican 18)	Culture is more important than religion in approaching ACP conversations (Anglican)	
Culturally a lot of the Muslim people, the non-practising ones believe once you close your eyes that’s it. The people that do practice the religion do believe there is after life and they’re going to heaven, to eternity. It all depends on the individual. Some people say I’m a Muslim. If somebody asks me my religion I’m Muslim, I practice the religion but I personally don’t believe in all the preaching’s of the Koran but I believe the principles – you should be a good citizen, love thy neighbour approach. (Islamic 14)	Range of cultural ways of living as a Muslim - non-practicing to practicing religion & belief in heaven/eternity; Practices the religion - doesn’t believe all Koran preachings but in good citizen principles	2b. Cultural traditions; types/sects within religions
Usually as you find with every culture and religion, a lot of religious expressions are cultural. So if I’m working with Buddhist people I’m not talking about Buddhists, I’m talking about the six different expressions of Buddhism so that makes it very complex. (Christian 02)	Buddhism encompasses different expressions of faith	
If you’re in tune with orthodox spirituality you’re happy to die, the dying process is a peaceful one. There is not that fear and angst around it. That’s a cultural phenomenon rather than – you see some very faithful people and their death is so smooth and easy. Then you see other people who aren’t, not that they aren’t faithful but they don’t follow the faith as much as others, they’re screaming, pulling their hair out, crying. That’s not the orthodox faith because we believe the person continues to live. (Greek Orthodox 20)	If in tune with Greek Orthodox spirituality, happy to die/ peaceful; less faithful scream, pull hair out, cry	2c. Personal attitudes and religiosity
I think these days the Lutheran church is kind of like a lot of churches in some ways, the rigid doctrine isn’t what’s expected by most and it’s not practiced by most. For some of them this sort of thing in some ways can be a bit hard to make connections. Okay a funeral yes, everybody has to have a funeral but to think of their life and their choices can be a little bit of a different. Again there is nothing prescribed within the Lutheran faith no matter where you might be on the spectrum – rigid, conservative, fairly liberal, haven’t really been involved very much at all. To me I think it will be much more of a personal thing. (Lutheran 16)	Lutheran - ‘like a lot of little churches’ Relationship with Lutheranism is personal, seldom strong	
Each case is different and you can’t say in this case the Rabbi said it’s okay and this case the Rabbi said it’s not okay they must be arguing. Not necessarily, maybe they have different scenarios. Say one scenario the health of the person has deteriorated – you have questions about whether to put a peg in to feed a person. You don’t want to starve a person but to what level do you feed them? These are all questions that come into the criteria. When you just have the RCA (Rabbinical Council of America) document it’s hard to get the information you need out of it that’s yes, no, black and white. That’s why I think it’s important the patient or some Rabbi should be involved in this process also. (Jewish 37)	Rabbi should be involved in deciding if tube fed, etc., as no black and white decisions	2d. Interpretations of faith and doctrine, and misunderstandings
There are people that come from kind of a Catholic heritage that mistakenly think they can’t put a not for resuscitation directive in their care plan because it’s goes against their faith teaching which is in fact not the case. So not for resuscitation is a perfectly acceptable – not to have this kind of extraordinary situation of people intervening when it’s clearly impossible for this person to be brought back, to be resuscitated. (Catholic 19)	Some Catholics think can’t put not for resuscitation as against faith (wrong)	

### Theme 1 religious leaders have diverse ACP understanding and experiences

#### 1a. Fragmented ACP understanding but general positive regard

Religious leaders mostly regarded ACP as a process of considering end-of-life care preferences but seldom mentioned the need to communicate them. Only seven indicated that ACPs were enacted when a person lost capacity and five had no ACP understanding.

[ACP is] I assume, that the medical system, whether it’s aged care or hospice system, they look after the patients to the extent where they’re able to do preventive measures, to have them in a comfortable state or also to allocate measures that will cure them of their illness as well. And to make their life comfortable. (Sikh 33).

When understood, most supported ACP because, for example, it helps preparation for death, and is a means of “communicating independence” when dependent. Several leaders were concerned that some could regard ACP as “indulgent” (Buddhist 04), or diverting from “quality of living” [to] “you’re dying” (Jewish Orthodox 05).

#### 1b. Engagement in followers’ ACP in various ways

Leaders’ descriptions of their own interactions with seriously ill patients could be described as ACP, although the term was seldom used. Many communicate with followers about their end-of-life concerns and facilitate actualisation of preferred spiritual care, including religious customs, scripture readings, or prayers. Even when leaders don’t engage in ACP conversations with patients, some are later consulted by families on religious implications of healthcare decision-making. For example, Coptic Orthodox religious leaders often support families who believe that “God is the one that is to take away the life and not us” (Coptic Orthodox 31), through challenging hospitals wanting to cease life support. Bahá’í local spiritual assemblies are only consulted on ACP if followers do not have family, but can advise families on post-death rituals. When patients had not discussed future care requirements with religious leaders, leaders may interpret followers’ unspoken wishes.

With the Greek Orthodox faith, it’s not in the lead up to the death that’s important to them. For some reason everything kicks in after they die. That’s when all the rituals kick in. There is a whole – we just follow it. (Greek Orthodox 27).

For Buddhists, this may be informed by considerations about a person’s name, origin, and place of worship.

### Theme 2 religious followers’ diverse approaches towards end-of-life care reflect varied religious and cultural backgrounds, and faith interpretations and attitudes

According to religious leaders, followers’ diverse approaches towards end-of-life and post-death planning (including avoidance of discussions) reflect their diverse backgrounds and attitudes as follows:

#### 2a. Family and geographical origins

Orthodox, Jewish, Hindu, Muslim, and Bahá’í leaders emphasised the family’s important role in ACP. Within the Asian Buddhist subcultures, close relatives manage ACP decisions and patients can have nil to intimate involvement. Time in Australia and/or elsewhere also affected faith expressions and degree of family involvement in end-of-life matters. A Rabbi (Jewish 13) stated there were “dozens of different views” within his congregation and many different mourning rituals depending on where people were born. Geographical origin could also affect information preferences, for example, those from Coptic Orthodox Egyptian backgrounds believe that too much prognostic information may do “more harm than good” (Coptic Orthodox 31), Russian Jews may not want older people to know bad prognoses, and Indian Hindu immigrants’ children should decide on information shared. Receiving bad news could have superstitious connotations for Chinese of all backgrounds.

Chinese are probably the strongest group who would want to protect the person themselves from knowing. Basically there is still quite a lot of superstition among quite well educated and Westernised Chinese around speaking of misfortune such as grave illness or death. That it’s kind of a no, no. (Buddhist 04)

#### 2b. Cultural traditions; types/sects within religions

Many followers’ faiths are tightly interwoven with cultural origins and identities.

The Bahá’í faith has many writings … and we try and be very faithful to the text. It is a text-based faith. However, the Bahá’ís come from very different cultural backgrounds and being human beings you’re always affected by the culture in which you’ve grown up. The Bahá’ís from Iran … (might have) more an Islamic perspective. But for those from a Western background we might have a more Christian way of looking at things. (Bahá’í 34).

A Greek Orthodox leader highlighted: “It’s the church that preserves the Greek identity, the Greek language, the Greek mindset” (Greek Orthodox 20).

#### 2c. Personal attitudes and religiosity

A spectrum of followers’ attitudes towards religions and ways of practising faith were observed by leaders.

You will get people who come out of Catholic heritage. Some will be on one end of the spectrum and say, “I don't give a rats about all of that”. Then you get the other end of the spectrum that say, “This is really important to me, whatever decisions and choices I make it needs to be consistent with my faith heritage”. (Catholic 19).

Attitudes could also change as patients approached death. A Muslim leader stated that often people who don’t practise all their life want to practise last rites, prayers, and recitations. Leaders’ personal preferences could also affect their approaches toward dying patients. A Buddhist Monk stated, “The one I personally like to do is the King of Prayers” (Buddhist 03).

#### 2d. Interpretation of faith and doctrine, and misunderstandings

Individuals may interpret faith principles in different ways, thereby making different decisions in the same ACP areas. The Bahá’í leader said that this reflected followers’ highly varied cultural backgrounds. Catholic and Jewish leaders also described both “progressive” and “fundamental” interpretations of doctrine. Healthcare decision-making reflecting faith-based values can also be difficult, notably amongst Catholic followers as they can struggle with what constitutes “ordinary means of preserving life,” as stated in doctrine:.

If someone is very concerned only about avoiding extraordinary means (of preserving life) they may miss the other boundary and go too far into refusing what is the ordinary means of preserving life. There might be some ... at the conservative end of the spectrum who might be so concerned about avoiding euthanasia by omission that they may end up choosing overly burdensome care. (Catholic 17).

Catholic followers may also occasionally mistakenly link ACP with euthanasia or assisted dying.

### Theme 3 religious expressions are multifaceted; health professionals need to enquire about what matters

#### 3a. General knowledge and communication competencies recommended; don’t generalise

Leaders recommended that health professionals recognise how religious/spiritual care may facilitate improved medical care, and acquire broad knowledge about religions, accompanying ways of living, and culturally sensitive, family-based communication skills.

[What] the health care professional must understand is the sensitivity to the person and a general understanding of the Hindu way of life. Not to an expert level but just to understand, yes they believe in rebirth and they believe what you do in this life is going to affect your future without complicating it further. That’s basic level of empathy will be I think good, very useful in planning at this time [sic]. (Hindu 24).

A Uniting Church leader however cautioned against a “cultural folder”, stating, for example, “All Muslims believe this or all Catholics believe that”, because they are “often incorrect in relation to the individual” (Uniting Church 01). Most leaders supported disclosure of bad prognoses, but suggested caution when people declined information offered.

#### 3b. Openness to individualised and/or standardised religious expressions

While occasional standardised religious expressions were associated with end-of-life care, such as Catholicism’s ‘Sacrament of the Sick’, Anglican, Baptist, Presbyterian, Uniting Church, and Lutheran religious leaders emphasised individualised approaches towards end-of-life religious rituals.

[End-of-life prayer is] not essential as a means of transition from this life to the next ... because they feel it’s already done. Jesus has done that so they have faith in Him. (Presbyterian 10).

One said, “For Baptists, there is no ‘have to’, it’s an individual’s decision what they want.” (Baptist 35). Anyone can read scripture when Sikh followers die. Further, religious expressions could be embedded in lived lives. A Jewish leader stated, “Belief is very personal ... there is no dogma” (Jewish 05) and a Hindu stated that, “…at the moment of dying there is no particular belief. The belief in total is that one should have [lived] a good life” (Hindu 26).

Many leaders recommended inquiring about meaningful issues in patients’ lives relevant to ACP. Some suggested asking about faith backgrounds, however, one leader (Coptic Orthodox 31) considered this could offend, preferring, “what beliefs and what values do you have?” (Further conversation suggestions see [57]).

Through these conversations, it was suggested, health professionals could gauge the importance of prayers, singing/chanting, Holy Communion, last rites, restrictions around bathing, touching and moving the deceased body, fasting, confession, and other rituals; and sensitivities around gender of those providing information, end-of-life treatment decisions, and pain management aims. A leader also warned against offending through religious based assumptions of individuals’ requirements, stating “We can’t assume anything” (Buddhist 04).

#### 3c. Successful ACP needs early consideration and improved community awareness

Many leaders, however, considered that ACP should commence before hospitalisation, with conversations initiated through general practitioners, public forums, everyday family conversations, and religious and neighbourhood communities.

I think most of this [ACP] process needs to happen in the community ... in community health centres, in neighbourhood houses, cultural groups, temples. ... so you may begin with the GP and then you’re taking [it] out into the community into a bigger context … Death cafes have been hugely successful because it opens up that opportunity for people. (Buddhist 04).

A Christian also suggested that religious leaders are no better than others at confronting issues about death.

## Discussion

Despite religious leaders often supporting patients and families in advanced illnesses [47], in an Australian-based cohort of religious leaders, most only had partial understanding of ACP. Once the concept was clarified religious leaders found it acceptable, a finding that is consistent with a group of Catholic Nuns [56]. The preparatory nature of ACP was expressed by leaders as a means of considering death when still capable therefore instilling a sense of control when dependent on others. Similar sentiments have been articulated by patients undergoing haemodialysis [51] and with human immunodeficiency virus [52].

Interrelated reasons were attributed to followers’ diverse approaches to ACP. Of note, a Jewish leader mentioned that Russian Jews may not want older people to know bad prognoses, and a Hindu leader stated that Indian Hindu immigrants’ children should make decisions regarding information sharing to patients. Findings are consistent with conflicting value assumptions and meanings expressed in relation to decision-making, truth telling and control over the dying process between western and other cultural worldviews [67]. This has implications for health professionals who have a medical, ethical and legal responsibility to provide patient care. Conducting culturally appropriate end-of-life decision-making conversations requires careful assessment and communication to ensure mutually acceptable goals between the health professional, patient and family members [68].

Religious leaders highlighted personal attitudes and level of religiosity as impacting decision-making preferences. Within religions, differences have been previously described where fundamentalist Catholics and Protestants preferred life-sustaining treatment compared to their non-fundamentalist counterparts [69]. Additionally, people with greater religiosity have preferred more life sustaining treatment [9, 16], with religious participants less likely to want euthanasia than those who were considered only affiliated [16]. Preferences for life sustaining treatment and euthanasia were not specifically explored in our study.

Bahá’í, Jewish and Catholic leaders, spoke about differences in interpretations of faith, doctrine and misunderstandings. Of note, a Catholic leader stated some followers may misconstrue ACP to be tied to euthanasia or assisted dying which may deter some followers from participation. Considering religious leaders’ diverse accounts of involvement in patients’ spiritual care, they could play a greater role in participating in ACP conversations by alleviating misconceptions related to their faith and ACP.

Religious expressions were multifaceted in our study with religious leaders emphasising the importance of religious and spiritual care to improve medical care. The need for health professionals to gain knowledge about religious beliefs surrounding death and dying was widely deemed as useful in preparation for ACP. Equally important was religious leaders’ caution against assumptions based on religion or culture and the emphasis to consult patients about what they considered appropriate, which has been articulated in the literature [70, 71].

Early initiation of ACP conversations was considered important by religious leaders, a finding consistent with general practitioners treating dementia patients [72]. Neighbourhood forums, faith communities and death cafes were mentioned as opportunities to discuss ACP. Religious leaders’ awareness of death cafes indicates their familiarity with the contemporary public initiatives [73, 74] which are aimed at normalising conversations about death and dying within the community. These may be plausible avenues for religious leaders to facilitate improved knowledge and awareness of ACP in their faith community.

### Strengths and limitations

This is the first study exploring how religious leaders, who represented diverse Australian-based faiths [75], understand ACP, and their perceptions of followers’ approaches to ACP and end-of-life care. An interesting finding was that religious leaders were participating in ACP without possibly recognising this. Findings may be useful to other countries that have similar multi-faith and multicultural populations, however given the heterogeneity within religions, there are likely to be differences among religious and cultural backgrounds. Lessons learnt therefore may have broader applicability to the United States, Canada and the United Kingdom, where like Australia, religious beliefs and values have been incorporated in national frameworks and guidelines [2, 3, 76, 77] addressing ACP and palliative care.

A limitation of our study is that religious leaders were recruited through contacts from the ACP*Talk* Project Advisory Committee, Sikh, Bahá’í and Lutheran religions were each represented by only one leader’s interview, and some religions were not represented. Other religious leaders may have additional and contrasting perspectives. Hence, although the findings were repetitive, they were not saturated. Nonetheless, the information provides valuable novel insights into how religious leaders participate in followers’ ACPs, explanations for why followers of same religions can vary in their approach to end-of-life care, and suggestions for how health professionals can sensitively approach ACP with people from different religious backgrounds. None of the religious leaders were known to the researchers conducting interviews or data analysis so this is unlikely to have biased results.

## Conclusion

Our study has shown that most religious leaders have some understanding of ACP. Religious beliefs and values around death and dying could be useful in preparing health professionals for ACP with patients of different religions. Equally important is the need for health professionals to provide culturally sensitive care without assumptions based on religion or culture. Religious leaders could play a greater role in ACP by clarifying followers’ faith misconceptions, encouraging patients to discuss the implications of their faith with health professionals and raising awareness of ACP within their communities. Further qualitative research could focus on how followers from different religious backgrounds consider that ACP could be optimally offered and assisted amongst members.

## Abbreviation

ACP: Advance care planning
